# Hexachloridobis­{*μ_2_*-2-(piperazin-1-yl)-*N*-[1-(2-pyrid­yl)ethyl­idene]ethanamine}­trizinc dihydrate

**DOI:** 10.1107/S1600536811027437

**Published:** 2011-07-13

**Authors:** Nura Suleiman Gwaram, Hamid Khaledi, Hapipah Mohd Ali

**Affiliations:** a Department of Chemistry, University of Malaya, 50603 Kuala Lumpur, Malaysia

## Abstract

In the trinuclear title compound, [Zn_3_Cl_6_(C_13_H_20_N_4_)_2_]·2H_2_O, each terminal Zn^II^ atom is coordinated by an N_3_ donor set from the Schiff base ligands and two Cl atoms in a distorted square-pyramidal geometry. The central Zn^II^ atom is tetra­hedrally coordinated by two piperazine N atoms from two Schiff base ligands and two Cl atoms. The piperazine rings adopt chair conformations. In the crystal structure, adjacent complex mol­ecules are linked into a three-dimensional network *via* N—H⋯O, C—H⋯Cl and C—H⋯O hydrogen bonds. The structure includes two water mol­ecules, one of which is disordered over two positions with occupancies of 0.753 (15) and 0.247 (15).

## Related literature

For related structures, see: Mukhopadhyay *et al.* (2003 ▶); Xu *et al.* (2008 ▶). For a description of the geometry of complexes with five-coordinate metal ions, see: Addison *et al.* (1984 ▶).
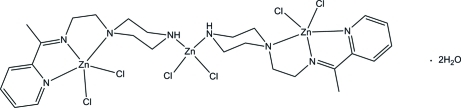


## Experimental

### 

#### Crystal data

[Zn_3_Cl_6_(C_13_H_20_N_4_)_2_]·2H_2_O
*M* *_r_* = 909.50
Triclinic, 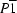

*a* = 7.6060 (3) Å
*b* = 14.8850 (5) Å
*c* = 16.7153 (5) Å
α = 72.570 (2)°
β = 86.834 (2)°
γ = 88.936 (2)°
*V* = 1802.78 (11) Å^3^
*Z* = 2
Mo *K*α radiation
μ = 2.46 mm^−1^
*T* = 100 K
0.21 × 0.12 × 0.09 mm

#### Data collection

Bruker APEXII CCD diffractometer
Absorption correction: multi-scan (*SADABS*; Sheldrick, 1996 ▶) *T*_min_ = 0.626, *T*_max_ = 0.809
16465 measured reflections
7831 independent reflections
6241 reflections with *I* > 2σ(*I*)
*R*_int_ = 0.024

#### Refinement

*R*[*F* ^2^ > 2σ(*F* ^2^)] = 0.046
*wR*(*F* ^2^) = 0.127
*S* = 1.04
7831 reflections
424 parameters
8 restraints
H atoms treated by a mixture of independent and constrained refinement
Δρ_max_ = 0.92 e Å^−3^
Δρ_min_ = −1.31 e Å^−3^

### 

Data collection: *APEX2* (Bruker, 2007 ▶); cell refinement: *SAINT* (Bruker, 2007 ▶); data reduction: *SAINT*; program(s) used to solve structure: *SHELXS97* (Sheldrick, 2008 ▶); program(s) used to refine structure: *SHELXL97* (Sheldrick, 2008 ▶); molecular graphics: *X-SEED* (Barbour, 2001 ▶); software used to prepare material for publication: *SHELXL97* and *publCIF* (Westrip, 2010 ▶).

## Supplementary Material

Crystal structure: contains datablock(s) I, New_Global_Publ_Block. DOI: 10.1107/S1600536811027437/ci5190sup1.cif

Structure factors: contains datablock(s) I. DOI: 10.1107/S1600536811027437/ci5190Isup2.hkl

Additional supplementary materials:  crystallographic information; 3D view; checkCIF report

## Figures and Tables

**Table 1:** Hydrogen-bond geometry (Å, °)

*D*—H⋯*A*	*D*—H	H⋯*A*	*D*⋯*A*	*D*—H⋯*A*
N4—H4*N*⋯O2^i^	0.91 (2)	2.05 (2)	2.953 (7)	170 (5)
N5—H5*N*⋯O1^i^	0.92 (2)	2.41 (2)	3.310 (6)	169 (5)
C3—H3⋯Cl6^ii^	0.95	2.78	3.563 (4)	140
C7—H7*C*⋯Cl6^iii^	0.98	2.79	3.634 (5)	144
C14—H14*A*⋯Cl3^iv^	0.99	2.78	3.493 (5)	130
C16—H16*B*⋯Cl6^v^	0.99	2.81	3.509 (5)	128
C19—H19*A*⋯Cl4^vi^	0.99	2.70	3.689 (6)	174
C11—H11*A*⋯Cl2	0.99	2.81	3.524 (5)	129
C13—H13*B*⋯Cl1	0.99	2.63	3.461 (5)	141
C17—H17*A*⋯Cl5	0.99	2.78	3.491 (5)	129
C14—H14*A*⋯O2^i^	0.99	2.59	3.331 (9)	131
C15—H15*B*⋯O1^i^	0.99	2.60	3.431 (7)	142

Symmetry codes: (i) 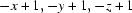; (ii) 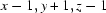; (iii) 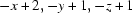; (iv) 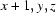; (v) 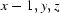; (vi) 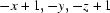.

**Fig. 1.**
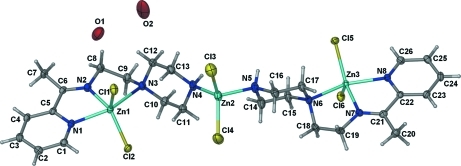
The molecular structure of the title compound (50% probability displacement ellipsoids). Only the major component of disordered O2 is shown.

## supplementary crystallographic information

### Comment

It has been shown that ligands having piperazine ring, depending on the ring conformation (chair or boat), may display ambidentate ligation behavior (Mukhopadhyay *et al.*, 2003; Xu *et al.*, 2008). The title compound was obtained upon the reaction of the *in situ* prepared Schiff base, 2-piperazino-*N*-[1-(2-pyridyl)ethylidene]ethanamine, with zinc(II) chloride. As shown in Fig. 1, the compound contains three unique metal centers. One Schiff base ligand chelates each of the two terminal zinc atoms *via* three N atoms. The piperazine rings assume chair conformations and their nitrogen atoms, N4 and N5, which stay away from chelation, are coordinated to the central zinc atom. Within the resulting trinuclear zinc complex, the metal atoms are separated by 5.8498 (7) Å (Zn1···Zn2) and 7.0153 (7) Å (Zn2···Zn3). The coordination environment around each metal center is completed by two Cl atoms, resulting in a tetrahedral geometry for the central Zn^II^ ion and square-pyramidal geometries for the terminal zinc atoms (τ = 0.39 and 0.26 for Zn1 and Zn3 centers, respectively, Addison *et al.*, 1984). The crystal structure contains intra- and intermolecular C—H···Cl interactions (Table 1). The latter connect the adjacent metal complexes into a three-dimensional network. The complex is N—H···O and C—H···O hydrogen bonded to two molecules of water, one of which being disordered between two sites.

### Experimental

A mixture of 4-(2-aminoethyl) piperazine (0.2 g, 1.55 mmol) and 2-acetylpyridine (0.19 g, 1.55 mmol) in ethanol (20 ml) was refluxed for 2 h and then an ethanolic solution of zinc (II) chloride (0.21 g, 1.55 mmol) was added. The resulting solution was refluxed for 30 min and then set aside at room temperature for a few days whereupon the prismatic crystals of the title compound were obtained.

### Refinement

The C-bound H atoms were placed at calculated positions and refined as riding on their parent atoms, with C–H = 0.95 (aryl), 0.98 (methyl) and 0.99 (methylene) Å. The N-bound H atoms were located in a difference Fourier map and refined with a N–H distance restraint of 0.91 (2) Å. For all H atoms *U*_iso_(H) were set to 1.2 (1.5 for methyl) times the *U*_eq_(carrier atom). H atoms of the water molecules could not be reliably located and were omitted from the refinement. One of the water oxygen atoms (O2) is disordered over two positions with refined site occupancy factors of 0.753 (15) and 0.247 (15). An *ISOR* restraint (Sheldrick, 2008) was applied to the major component of the disordered water oxygen atom (O2).

### Crystal data

**Table:**

[Zn_3_Cl_6_(C_13_H_20_N_4_)_2_]·2H_2_O	*Z* = 2
*M**_r_* = 909.50	*F*(000) = 928
Triclinic, *P*1	*D*_x_ = 1.675 Mg m^−^^3^
Hall symbol: -P 1	Mo *K*α radiation, λ = 0.71073 Å
*a* = 7.6060 (3) Å	Cell parameters from 5640 reflections
*b* = 14.8850 (5) Å	θ = 2.6–29.0°
*c* = 16.7153 (5) Å	µ = 2.46 mm^−^^1^
α = 72.570 (2)°	*T* = 100 K
β = 86.834 (2)°	Prism, yellow
γ = 88.936 (2)°	0.21 × 0.12 × 0.09 mm
*V* = 1802.78 (11) Å^3^	

### Data collection

**Table:**

Bruker APEXII CCD diffractometer	7831 independent reflections
Radiation source: fine-focus sealed tube	6241 reflections with *I* > 2σ(*I*)
graphite	*R*_int_ = 0.024
φ and ω scans	θ_max_ = 27.0°, θ_min_ = 2.2°
Absorption correction: multi-scan (*SADABS*; Sheldrick, 1996)	*h* = −9→9
*T*_min_ = 0.626, *T*_max_ = 0.809	*k* = −18→19
16465 measured reflections	*l* = −21→21

### Refinement

**Table:**

Refinement on *F*^2^	Primary atom site location: structure-invariant direct methods
Least-squares matrix: full	Secondary atom site location: difference Fourier map
*R*[*F*^2^ > 2σ(*F*^2^)] = 0.046	Hydrogen site location: inferred from neighbouring sites
*wR*(*F*^2^) = 0.127	H atoms treated by a mixture of independent and constrained refinement
*S* = 1.04	*w* = 1/[σ^2^(*F*_o_^2^) + (0.0573*P*)^2^ + 5.66*P*] where *P* = (*F*_o_^2^ + 2*F*_c_^2^)/3
7831 reflections	(Δ/σ)_max_ = 0.001
424 parameters	Δρ_max_ = 0.92 e Å^−^^3^
8 restraints	Δρ_min_ = −1.31 e Å^−^^3^

### Special details

**Table:**

Geometry. All e.s.d.'s (except the e.s.d. in the dihedral angle between two l.s. planes) are estimated using the full covariance matrix. The cell e.s.d.'s are taken into account individually in the estimation of e.s.d.'s in distances, angles and torsion angles; correlations between e.s.d.'s in cell parameters are only used when they are defined by crystal symmetry. An approximate (isotropic) treatment of cell e.s.d.'s is used for estimating e.s.d.'s involving l.s. planes.
Refinement. Refinement of *F*^2^ against ALL reflections. The weighted *R*-factor *wR* and goodness of fit *S* are based on *F*^2^, conventional *R*-factors *R* are based on *F*, with *F* set to zero for negative *F*^2^. The threshold expression of *F*^2^ > σ(*F*^2^) is used only for calculating *R*-factors(gt) *etc*. and is not relevant to the choice of reflections for refinement. *R*-factors based on *F*^2^ are statistically about twice as large as those based on *F*, and *R*- factors based on ALL data will be even larger.

### Fractional atomic coordinates and isotropic or equivalent isotropic displacement parameters (Å^2^)

**Table:**

	*x*	*y*	*z*	*U*_iso_*/*U*_eq_	Occ. (<1)
Zn1	0.60721 (6)	0.60302 (3)	0.15462 (3)	0.01962 (12)	
Zn2	0.54211 (8)	0.26399 (4)	0.44315 (3)	0.03191 (15)	
Zn3	0.96674 (6)	−0.03792 (3)	0.78382 (3)	0.01865 (12)	
Cl1	0.32182 (14)	0.57174 (8)	0.20310 (7)	0.0286 (2)	
Cl2	0.76455 (14)	0.49402 (7)	0.11373 (6)	0.0245 (2)	
Cl3	0.25702 (18)	0.28147 (10)	0.47790 (9)	0.0461 (3)	
Cl4	0.6177 (2)	0.18718 (10)	0.35034 (8)	0.0451 (3)	
Cl5	0.82202 (14)	0.06069 (7)	0.84965 (6)	0.0251 (2)	
Cl6	1.24603 (14)	0.01080 (8)	0.74837 (7)	0.0276 (2)	
N1	0.5433 (4)	0.6860 (2)	0.0275 (2)	0.0191 (7)	
N2	0.6720 (5)	0.7394 (2)	0.1477 (2)	0.0213 (7)	
N3	0.7470 (5)	0.5792 (2)	0.2849 (2)	0.0222 (7)	
N4	0.6610 (5)	0.3943 (3)	0.4067 (2)	0.0279 (8)	
H4N	0.724 (6)	0.400 (4)	0.450 (2)	0.033*	
N5	0.6552 (5)	0.1858 (3)	0.5515 (2)	0.0302 (9)	
H5N	0.657 (7)	0.210 (3)	0.596 (2)	0.036*	
N6	0.8522 (5)	0.0241 (3)	0.6575 (2)	0.0245 (8)	
N7	0.9023 (5)	−0.1581 (2)	0.7520 (2)	0.0219 (7)	
N8	1.0000 (4)	−0.1532 (2)	0.8977 (2)	0.0178 (7)	
C1	0.4820 (6)	0.6548 (3)	−0.0321 (3)	0.0226 (9)	
H1	0.4640	0.5891	−0.0205	0.027*	
C2	0.4431 (6)	0.7137 (3)	−0.1103 (3)	0.0236 (9)	
H2	0.3980	0.6890	−0.1512	0.028*	
C3	0.4711 (6)	0.8092 (3)	−0.1279 (3)	0.0239 (9)	
H3	0.4465	0.8515	−0.1812	0.029*	
C4	0.5359 (6)	0.8422 (3)	−0.0658 (3)	0.0217 (8)	
H4	0.5566	0.9075	−0.0762	0.026*	
C5	0.5702 (5)	0.7791 (3)	0.0112 (2)	0.0186 (8)	
C6	0.6400 (5)	0.8070 (3)	0.0824 (3)	0.0190 (8)	
C7	0.6619 (7)	0.9095 (3)	0.0729 (3)	0.0273 (10)	
H7A	0.5463	0.9377	0.0788	0.041*	
H7B	0.7166	0.9411	0.0174	0.041*	
H7C	0.7370	0.9167	0.1164	0.041*	
C8	0.7424 (7)	0.7540 (3)	0.2227 (3)	0.0275 (10)	
H8A	0.6448	0.7634	0.2612	0.033*	
H8B	0.8182	0.8106	0.2067	0.033*	
C9	0.8485 (6)	0.6676 (3)	0.2656 (3)	0.0273 (10)	
H9A	0.9531	0.6636	0.2288	0.033*	
H9B	0.8906	0.6745	0.3184	0.033*	
C10	0.8763 (6)	0.5001 (3)	0.3062 (3)	0.0265 (9)	
H10A	0.9482	0.5061	0.3519	0.032*	
H10B	0.9566	0.5042	0.2565	0.032*	
C11	0.7864 (6)	0.4050 (3)	0.3335 (3)	0.0266 (9)	
H11A	0.7224	0.3970	0.2861	0.032*	
H11B	0.8768	0.3549	0.3480	0.032*	
C12	0.6261 (6)	0.5688 (3)	0.3592 (3)	0.0274 (10)	
H12A	0.5362	0.6193	0.3459	0.033*	
H12B	0.6933	0.5762	0.4059	0.033*	
C13	0.5352 (6)	0.4741 (3)	0.3870 (3)	0.0286 (10)	
H13A	0.4573	0.4701	0.4373	0.034*	
H13B	0.4606	0.4689	0.3419	0.034*	
C14	0.8469 (7)	0.1730 (4)	0.5366 (3)	0.0367 (12)	
H14A	0.9034	0.2355	0.5142	0.044*	
H14B	0.8655	0.1394	0.4939	0.044*	
C15	0.9338 (6)	0.1179 (3)	0.6160 (3)	0.0321 (11)	
H15A	1.0598	0.1088	0.6020	0.039*	
H15B	0.9274	0.1553	0.6560	0.039*	
C16	0.5725 (6)	0.0910 (3)	0.5870 (3)	0.0304 (10)	
H16A	0.5846	0.0562	0.5449	0.036*	
H16B	0.4453	0.0985	0.5995	0.036*	
C17	0.6584 (6)	0.0347 (3)	0.6668 (3)	0.0271 (9)	
H17A	0.6317	0.0658	0.7108	0.033*	
H17B	0.6048	−0.0288	0.6865	0.033*	
C18	0.9066 (7)	−0.0458 (3)	0.6135 (3)	0.0340 (11)	
H18A	1.0359	−0.0432	0.6025	0.041*	
H18B	0.8510	−0.0298	0.5588	0.041*	
C19	0.8535 (7)	−0.1444 (3)	0.6655 (3)	0.0319 (11)	
H19A	0.7249	−0.1527	0.6641	0.038*	
H19B	0.9146	−0.1913	0.6425	0.038*	
C20	0.8598 (6)	−0.3303 (3)	0.7939 (3)	0.0281 (10)	
H20A	0.8195	−0.3189	0.7371	0.042*	
H20B	0.7658	−0.3608	0.8350	0.042*	
H20C	0.9638	−0.3713	0.8013	0.042*	
C21	0.9060 (5)	−0.2388 (3)	0.8068 (3)	0.0205 (8)	
C22	0.9658 (5)	−0.2394 (3)	0.8906 (2)	0.0175 (8)	
C23	0.9890 (5)	−0.3205 (3)	0.9555 (3)	0.0217 (8)	
H23	0.9601	−0.3801	0.9499	0.026*	
C24	1.0555 (6)	−0.3137 (3)	1.0295 (3)	0.0243 (9)	
H24	1.0755	−0.3689	1.0746	0.029*	
C25	1.0917 (5)	−0.2263 (3)	1.0365 (3)	0.0225 (9)	
H25	1.1370	−0.2201	1.0864	0.027*	
C26	1.0607 (5)	−0.1473 (3)	0.9690 (3)	0.0216 (8)	
H26	1.0838	−0.0868	0.9742	0.026*	
O1	0.2839 (6)	0.7374 (3)	0.2857 (3)	0.0589 (12)	
O2	0.1410 (14)	0.6105 (5)	0.4450 (5)	0.087 (3)	0.753 (15)
O2'	0.007 (3)	0.5754 (12)	0.4953 (12)	0.053 (8)	0.247 (15)

### Atomic displacement parameters (Å^2^)

**Table:**

	*U*^11^	*U*^22^	*U*^33^	*U*^12^	*U*^13^	*U*^23^
Zn1	0.0219 (3)	0.0135 (2)	0.0220 (2)	−0.00288 (18)	−0.00124 (19)	−0.00282 (18)
Zn2	0.0386 (3)	0.0274 (3)	0.0255 (3)	−0.0038 (2)	−0.0047 (2)	−0.0007 (2)
Zn3	0.0221 (2)	0.0161 (2)	0.0160 (2)	−0.00138 (18)	0.00034 (18)	−0.00237 (17)
Cl1	0.0213 (5)	0.0296 (6)	0.0287 (5)	−0.0036 (4)	−0.0022 (4)	0.0009 (4)
Cl2	0.0298 (6)	0.0179 (5)	0.0266 (5)	0.0028 (4)	−0.0043 (4)	−0.0074 (4)
Cl3	0.0356 (7)	0.0509 (8)	0.0476 (8)	−0.0113 (6)	−0.0041 (6)	−0.0075 (6)
Cl4	0.0651 (9)	0.0370 (7)	0.0349 (7)	−0.0077 (6)	−0.0039 (6)	−0.0127 (5)
Cl5	0.0317 (6)	0.0201 (5)	0.0239 (5)	0.0007 (4)	0.0017 (4)	−0.0079 (4)
Cl6	0.0233 (5)	0.0270 (5)	0.0275 (5)	−0.0015 (4)	0.0008 (4)	−0.0009 (4)
N1	0.0188 (17)	0.0145 (16)	0.0227 (17)	−0.0017 (13)	−0.0006 (14)	−0.0034 (13)
N2	0.0224 (18)	0.0176 (17)	0.0236 (18)	−0.0029 (14)	−0.0039 (14)	−0.0050 (14)
N3	0.0266 (19)	0.0172 (17)	0.0217 (17)	−0.0053 (14)	−0.0024 (14)	−0.0035 (14)
N4	0.032 (2)	0.025 (2)	0.0221 (18)	−0.0021 (16)	−0.0055 (16)	0.0000 (15)
N5	0.029 (2)	0.031 (2)	0.0248 (19)	0.0015 (17)	−0.0018 (16)	0.0010 (16)
N6	0.0255 (19)	0.0244 (19)	0.0184 (17)	0.0008 (15)	0.0018 (14)	0.0008 (14)
N7	0.0265 (19)	0.0231 (18)	0.0180 (17)	0.0014 (15)	−0.0047 (14)	−0.0084 (14)
N8	0.0173 (17)	0.0176 (17)	0.0172 (16)	−0.0018 (13)	0.0001 (13)	−0.0032 (13)
C1	0.024 (2)	0.020 (2)	0.023 (2)	−0.0011 (17)	0.0003 (17)	−0.0058 (16)
C2	0.020 (2)	0.028 (2)	0.024 (2)	−0.0028 (17)	−0.0017 (17)	−0.0084 (17)
C3	0.022 (2)	0.022 (2)	0.023 (2)	0.0003 (17)	0.0011 (17)	−0.0013 (17)
C4	0.023 (2)	0.0137 (19)	0.026 (2)	−0.0008 (16)	0.0028 (17)	−0.0025 (16)
C5	0.0151 (19)	0.0168 (19)	0.022 (2)	0.0001 (15)	0.0036 (15)	−0.0040 (15)
C6	0.0170 (19)	0.0138 (19)	0.025 (2)	−0.0009 (15)	0.0023 (16)	−0.0044 (15)
C7	0.041 (3)	0.014 (2)	0.026 (2)	−0.0017 (18)	−0.0008 (19)	−0.0035 (17)
C8	0.041 (3)	0.016 (2)	0.027 (2)	−0.0053 (18)	−0.0080 (19)	−0.0063 (17)
C9	0.029 (2)	0.025 (2)	0.028 (2)	−0.0038 (18)	−0.0091 (18)	−0.0055 (18)
C10	0.025 (2)	0.024 (2)	0.030 (2)	0.0022 (18)	−0.0046 (18)	−0.0060 (18)
C11	0.030 (2)	0.023 (2)	0.025 (2)	0.0012 (18)	−0.0010 (18)	−0.0049 (17)
C12	0.035 (3)	0.026 (2)	0.021 (2)	0.0035 (19)	−0.0014 (18)	−0.0073 (17)
C13	0.029 (2)	0.029 (2)	0.024 (2)	−0.0008 (19)	0.0014 (18)	−0.0037 (18)
C14	0.032 (3)	0.036 (3)	0.030 (2)	0.001 (2)	0.003 (2)	0.006 (2)
C15	0.024 (2)	0.034 (3)	0.027 (2)	−0.0012 (19)	0.0006 (18)	0.0079 (19)
C16	0.031 (2)	0.030 (2)	0.025 (2)	−0.005 (2)	0.0005 (19)	−0.0008 (19)
C17	0.025 (2)	0.027 (2)	0.024 (2)	−0.0038 (18)	0.0009 (17)	0.0003 (18)
C18	0.047 (3)	0.036 (3)	0.016 (2)	0.009 (2)	−0.0018 (19)	−0.0036 (18)
C19	0.046 (3)	0.034 (3)	0.021 (2)	0.008 (2)	−0.008 (2)	−0.0153 (19)
C20	0.031 (2)	0.021 (2)	0.036 (2)	0.0013 (18)	−0.0056 (19)	−0.0131 (19)
C21	0.0155 (19)	0.022 (2)	0.024 (2)	0.0014 (16)	−0.0010 (16)	−0.0082 (16)
C22	0.0154 (19)	0.0169 (19)	0.0194 (19)	0.0000 (15)	0.0011 (15)	−0.0045 (15)
C23	0.021 (2)	0.0170 (19)	0.026 (2)	0.0000 (16)	0.0019 (16)	−0.0043 (16)
C24	0.023 (2)	0.022 (2)	0.022 (2)	0.0027 (17)	−0.0004 (17)	0.0014 (17)
C25	0.019 (2)	0.029 (2)	0.018 (2)	−0.0014 (17)	−0.0002 (16)	−0.0044 (17)
C26	0.021 (2)	0.022 (2)	0.022 (2)	−0.0041 (16)	0.0002 (16)	−0.0067 (16)
O1	0.061 (3)	0.048 (3)	0.072 (3)	0.017 (2)	−0.023 (2)	−0.022 (2)
O2	0.139 (7)	0.064 (4)	0.064 (5)	−0.018 (4)	−0.060 (5)	−0.016 (3)
O2'	0.082 (16)	0.034 (10)	0.048 (12)	−0.001 (9)	−0.041 (11)	−0.014 (8)

### Geometric parameters (Å, °)

**Table:**

Zn1—N2	2.066 (3)	C7—H7B	0.98
Zn1—N1	2.190 (3)	C7—H7C	0.98
Zn1—Cl2	2.2436 (11)	C8—C9	1.514 (6)
Zn1—Cl1	2.2809 (11)	C8—H8A	0.99
Zn1—N3	2.404 (3)	C8—H8B	0.99
Zn2—N5	2.061 (4)	C9—H9A	0.99
Zn2—N4	2.061 (4)	C9—H9B	0.99
Zn2—Cl4	2.2325 (15)	C10—C11	1.514 (6)
Zn2—Cl3	2.2445 (15)	C10—H10A	0.99
Zn3—N7	2.086 (3)	C10—H10B	0.99
Zn3—N8	2.170 (3)	C11—H11A	0.99
Zn3—Cl6	2.2473 (12)	C11—H11B	0.99
Zn3—N6	2.253 (3)	C12—C13	1.513 (6)
Zn3—Cl5	2.3132 (11)	C12—H12A	0.99
N1—C1	1.328 (5)	C12—H12B	0.99
N1—C5	1.348 (5)	C13—H13A	0.99
N2—C6	1.276 (5)	C13—H13B	0.99
N2—C8	1.465 (5)	C14—C15	1.514 (6)
N3—C12	1.475 (6)	C14—H14A	0.99
N3—C9	1.479 (5)	C14—H14B	0.99
N3—C10	1.492 (6)	C15—H15A	0.99
N4—C11	1.480 (6)	C15—H15B	0.99
N4—C13	1.481 (6)	C16—C17	1.522 (6)
N4—H4N	0.910 (19)	C16—H16A	0.99
N5—C14	1.484 (6)	C16—H16B	0.99
N5—C16	1.492 (6)	C17—H17A	0.99
N5—H5N	0.916 (19)	C17—H17B	0.99
N6—C18	1.484 (6)	C18—C19	1.514 (7)
N6—C17	1.484 (6)	C18—H18A	0.99
N6—C15	1.490 (6)	C18—H18B	0.99
N7—C21	1.275 (5)	C19—H19A	0.99
N7—C19	1.467 (5)	C19—H19B	0.99
N8—C26	1.331 (5)	C20—C21	1.493 (6)
N8—C22	1.354 (5)	C20—H20A	0.98
C1—C2	1.383 (6)	C20—H20B	0.98
C1—H1	0.95	C20—H20C	0.98
C2—C3	1.381 (6)	C21—C22	1.494 (6)
C2—H2	0.95	C22—C23	1.376 (6)
C3—C4	1.389 (6)	C23—C24	1.393 (6)
C3—H3	0.95	C23—H23	0.95
C4—C5	1.381 (6)	C24—C25	1.374 (6)
C4—H4	0.95	C24—H24	0.95
C5—C6	1.499 (6)	C25—C26	1.391 (6)
C6—C7	1.497 (5)	C25—H25	0.95
C7—H7A	0.98	C26—H26	0.95
			
N2—Zn1—N1	75.73 (13)	H8A—C8—H8B	108.4
N2—Zn1—Cl2	130.04 (11)	N3—C9—C8	113.1 (4)
N1—Zn1—Cl2	95.06 (9)	N3—C9—H9A	109.0
N2—Zn1—Cl1	110.48 (11)	C8—C9—H9A	109.0
N1—Zn1—Cl1	95.26 (9)	N3—C9—H9B	109.0
Cl2—Zn1—Cl1	119.30 (4)	C8—C9—H9B	109.0
N2—Zn1—N3	78.63 (13)	H9A—C9—H9B	107.8
N1—Zn1—N3	153.39 (12)	N3—C10—C11	111.9 (4)
Cl2—Zn1—N3	95.99 (9)	N3—C10—H10A	109.2
Cl1—Zn1—N3	100.26 (9)	C11—C10—H10A	109.2
N5—Zn2—N4	107.07 (16)	N3—C10—H10B	109.2
N5—Zn2—Cl4	103.95 (13)	C11—C10—H10B	109.2
N4—Zn2—Cl4	109.56 (12)	H10A—C10—H10B	107.9
N5—Zn2—Cl3	106.53 (12)	N4—C11—C10	112.4 (4)
N4—Zn2—Cl3	108.81 (12)	N4—C11—H11A	109.1
Cl4—Zn2—Cl3	120.10 (6)	C10—C11—H11A	109.1
N7—Zn3—N8	75.48 (13)	N4—C11—H11B	109.1
N7—Zn3—Cl6	114.52 (11)	C10—C11—H11B	109.1
N8—Zn3—Cl6	101.42 (9)	H11A—C11—H11B	107.9
N7—Zn3—N6	77.89 (13)	N3—C12—C13	112.1 (4)
N8—Zn3—N6	151.58 (13)	N3—C12—H12A	109.2
Cl6—Zn3—N6	98.48 (10)	C13—C12—H12A	109.2
N7—Zn3—Cl5	135.93 (11)	N3—C12—H12B	109.2
N8—Zn3—Cl5	95.60 (9)	C13—C12—H12B	109.2
Cl6—Zn3—Cl5	109.54 (4)	H12A—C12—H12B	107.9
N6—Zn3—Cl5	96.62 (10)	N4—C13—C12	112.7 (4)
C1—N1—C5	118.7 (3)	N4—C13—H13A	109.1
C1—N1—Zn1	127.6 (3)	C12—C13—H13A	109.1
C5—N1—Zn1	113.7 (3)	N4—C13—H13B	109.1
C6—N2—C8	122.9 (4)	C12—C13—H13B	109.1
C6—N2—Zn1	120.1 (3)	H13A—C13—H13B	107.8
C8—N2—Zn1	116.9 (3)	N5—C14—C15	112.2 (4)
C12—N3—C9	110.6 (3)	N5—C14—H14A	109.2
C12—N3—C10	107.7 (3)	C15—C14—H14A	109.2
C9—N3—C10	107.0 (3)	N5—C14—H14B	109.2
C12—N3—Zn1	115.3 (3)	C15—C14—H14B	109.2
C9—N3—Zn1	100.3 (2)	H14A—C14—H14B	107.9
C10—N3—Zn1	115.5 (3)	N6—C15—C14	113.5 (4)
C11—N4—C13	108.7 (3)	N6—C15—H15A	108.9
C11—N4—Zn2	111.6 (3)	C14—C15—H15A	108.9
C13—N4—Zn2	113.8 (3)	N6—C15—H15B	108.9
C11—N4—H4N	107 (3)	C14—C15—H15B	108.9
C13—N4—H4N	106 (3)	H15A—C15—H15B	107.7
Zn2—N4—H4N	109 (3)	N5—C16—C17	111.1 (4)
C14—N5—C16	108.5 (4)	N5—C16—H16A	109.4
C14—N5—Zn2	111.3 (3)	C17—C16—H16A	109.4
C16—N5—Zn2	112.2 (3)	N5—C16—H16B	109.4
C14—N5—H5N	100 (4)	C17—C16—H16B	109.4
C16—N5—H5N	105 (3)	H16A—C16—H16B	108.0
Zn2—N5—H5N	118 (3)	N6—C17—C16	114.6 (4)
C18—N6—C17	113.5 (4)	N6—C17—H17A	108.6
C18—N6—C15	111.8 (4)	C16—C17—H17A	108.6
C17—N6—C15	109.7 (3)	N6—C17—H17B	108.6
C18—N6—Zn3	102.0 (3)	C16—C17—H17B	108.6
C17—N6—Zn3	110.7 (2)	H17A—C17—H17B	107.6
C15—N6—Zn3	108.9 (3)	N6—C18—C19	111.1 (4)
C21—N7—C19	122.9 (4)	N6—C18—H18A	109.4
C21—N7—Zn3	120.1 (3)	C19—C18—H18A	109.4
C19—N7—Zn3	117.0 (3)	N6—C18—H18B	109.4
C26—N8—C22	118.7 (3)	C19—C18—H18B	109.4
C26—N8—Zn3	126.7 (3)	H18A—C18—H18B	108.0
C22—N8—Zn3	114.4 (3)	N7—C19—C18	108.2 (4)
N1—C1—C2	123.0 (4)	N7—C19—H19A	110.1
N1—C1—H1	118.5	C18—C19—H19A	110.1
C2—C1—H1	118.5	N7—C19—H19B	110.1
C3—C2—C1	118.7 (4)	C18—C19—H19B	110.1
C3—C2—H2	120.6	H19A—C19—H19B	108.4
C1—C2—H2	120.6	C21—C20—H20A	109.5
C2—C3—C4	118.5 (4)	C21—C20—H20B	109.5
C2—C3—H3	120.7	H20A—C20—H20B	109.5
C4—C3—H3	120.7	C21—C20—H20C	109.5
C5—C4—C3	119.5 (4)	H20A—C20—H20C	109.5
C5—C4—H4	120.3	H20B—C20—H20C	109.5
C3—C4—H4	120.3	N7—C21—C20	126.1 (4)
N1—C5—C4	121.6 (4)	N7—C21—C22	115.3 (4)
N1—C5—C6	114.6 (3)	C20—C21—C22	118.5 (4)
C4—C5—C6	123.8 (4)	N8—C22—C23	122.0 (4)
N2—C6—C7	125.5 (4)	N8—C22—C21	114.6 (3)
N2—C6—C5	115.7 (4)	C23—C22—C21	123.4 (4)
C7—C6—C5	118.8 (3)	C22—C23—C24	118.9 (4)
C6—C7—H7A	109.5	C22—C23—H23	120.6
C6—C7—H7B	109.5	C24—C23—H23	120.6
H7A—C7—H7B	109.5	C25—C24—C23	119.2 (4)
C6—C7—H7C	109.5	C25—C24—H24	120.4
H7A—C7—H7C	109.5	C23—C24—H24	120.4
H7B—C7—H7C	109.5	C24—C25—C26	118.7 (4)
N2—C8—C9	107.9 (4)	C24—C25—H25	120.6
N2—C8—H8A	110.1	C26—C25—H25	120.6
C9—C8—H8A	110.1	N8—C26—C25	122.4 (4)
N2—C8—H8B	110.1	N8—C26—H26	118.8
C9—C8—H8B	110.1	C25—C26—H26	118.8

### Hydrogen-bond geometry (Å, °)

**Table:**

*D*—H···*A*	*D*—H	H···*A*	*D*···*A*	*D*—H···*A*
N4—H4N···O2^i^	0.91 (2)	2.05 (2)	2.953 (7)	170 (5)
N5—H5N···O1^i^	0.92 (2)	2.41 (2)	3.310 (6)	169 (5)
C3—H3···Cl6^ii^	0.95	2.78	3.563 (4)	140
C7—H7C···Cl6^iii^	0.98	2.79	3.634 (5)	144
C14—H14A···Cl3^iv^	0.99	2.78	3.493 (5)	130
C16—H16B···Cl6^v^	0.99	2.81	3.509 (5)	128
C19—H19A···Cl4^vi^	0.99	2.70	3.689 (6)	174
C11—H11A···Cl2	0.99	2.81	3.524 (5)	129
C13—H13B···Cl1	0.99	2.63	3.461 (5)	141
C17—H17A···Cl5	0.99	2.78	3.491 (5)	129
C14—H14A···O2^i^	0.99	2.59	3.331 (9)	131
C15—H15B···O1^i^	0.99	2.60	3.431 (7)	142

Symmetry codes: (i) −*x*+1, −*y*+1, −*z*+1; (ii) *x*−1, *y*+1, *z*−1; (iii) −*x*+2, −*y*+1, −*z*+1; (iv) *x*+1, *y*, *z*; (v) *x*−1, *y*, *z*; (vi) −*x*+1, −*y*, −*z*+1.

## References

[bb1] Addison, A. W., Rao, T. N., Reedijk, J., Rijn, V. J. & Verschoor, G. C. (1984). *J. Chem. Soc. Dalton Trans.* pp. 1349–1356.

[bb2] Barbour, L. J. (2001). *J. Supramol. Chem*, **1**, 189–191.

[bb3] Bruker (2007). *APEX2* and *SAINT* Bruker AXS Inc., Madison, Wisconsin, USA.

[bb4] Mukhopadhyay, S., Mandal, D., Ghosh, D., Goldberg, I. & Chaudhury, M. (2003). *Inorg. Chem.* **42**, 8439–8445. 10.1021/ic0346174 14658897

[bb5] Sheldrick, G. M. (1996). *SADABS* University of Göttingen, Germany.

[bb6] Sheldrick, G. M. (2008). *Acta Cryst.* A**64**, 112–122. 10.1107/S0108767307043930 18156677

[bb7] Westrip, S. P. (2010). *J. Appl. Cryst.* **43**, 920–925.

[bb8] Xu, R.-B., Xu, X.-Y., Wang, M.-Y., Wang, D.-Q., Yin, T., Xu, G.-X., Yang, X.-J., Lu, L.-D., Wang, X. & Lei, Y.-J. (2008). *J. Coord. Chem.* **61**, 3306–3313.

